# ‘I Call Them Our Village’: Experiences Of Caregivers in Ontario's Extensive Needs Service

**DOI:** 10.1111/cch.70347

**Published:** 2026-09-10

**Authors:** Victoria Rombos, Zainab O. Salami, Nicholas Denomey, Genevieve Ferguson, Renee Baysarowich, Dennis Newhook, Stephanie Sutherland, Irene Drmic, Jordan Edwards, Srishti Sharma, Kathryn Decker, Tamara Milicevic, Melanie Penner

**Affiliations:** ^1^ Holland Bloorview Kids Rehabilitation Hospital Toronto Ontario Canada; ^2^ Children's Hospital of Eastern Ontario Ottawa Ontario Canada; ^3^ Hamilton Health Sciences Hamilton Ontario Canada; ^4^ University of Toronto Toronto Ontario Canada

**Keywords:** caregiver experience, interdisciplinary care, mental health, neurodevelopment, personalized care, qualitative research

## Abstract

**Background:**

Children and youth with complex needs, including neurodevelopmental disabilities or acquired brain injuries and co‐occurring mental health and physical health diagnoses, often experience difficulties accessing care that they need. Three Ontario children's hospitals collaborated to form the Extensive Needs Service (ENS), a novel interdisciplinary programme that provides wraparound services for this population. Caregiver perspectives are essential to understand the child‐ and family‐level benefits and experiences of interdisciplinary programmes such as the ENS.

**Objective:**

The objective of this study was to explore the experiences that caregivers had while their child participated in the ENS.

**Methods:**

We used a qualitative descriptive design from a constructivist paradigm. Purposive sampling was used to garner diverse representation in child gender, caregiver race and/or ethnicity, socio‐economic status and ENS site. The interview guide was developed to evaluate key programme domains including family functioning, goal achievement, quality of life, service use and satisfaction. Individual, semi‐structured interviews were conducted, transcribed verbatim and analysed by multiple coders using reflexive thematic analysis.

**Results:**

We interviewed 29 caregivers with diverse representation across sites and demographic characteristics. We identified five key themes: (1) inadequate care experiences prior to the ENS; (2) met and unmet expectations of the ENS; (3) benefits of the ENS for the whole family; (4) the power of big and small changes; and (5) reluctant but ready to leave the programme.

**Conclusion:**

Caregivers entered the ENS programme after repeated inadequate care experiences. In stark contrast, they found that the ENS met or exceeded their expectations and provided tangible benefits through openness, adaptability, compassion and championing the autonomy of the child and family. Upon discharge, caregivers felt emboldened to better support their child and to manage day‐to‐day challenges. Programmes across the continuum of care should adopt similar principles of personalized, compassionate care to support children with complex needs and their families.

## Summary

- ENS is highly valued by families due to its personalized and family‐centred approach, the amount of support from clinical teams and the level of care for the whole family.
- The perspectives of people with lived experience should be included when designing and refining programmes to ensure that services reflect the needs of the population they are supporting.
- Future research should evaluate the impacts of personalized, wraparound care approaches on clinical outcomes for children and youth with extensive needs

## 1 Background

Children and youth with complex needs, including acquired brain injuries and neurodevelopmental conditions, experience difficulties accessing appropriate services (McGill et al. 2006; Wodehouse and McGill 2009). Oftentimes, their neurodevelopmental needs intersect with social complexities (McConnell et al. 2014) and co‐occurring developmental, mental and physical health conditions (Lai et al. 2019). Novel, wraparound approaches tailored to meet this population's complex and varied needs are required, but few approaches have yet been described in the literature. Ontario's Extensive Needs Service (ENS) is an innovative, interdisciplinary programme for children and youth with complex intersecting needs delivered across multiple sites (Rombos et al. 2026). Evaluation of the ENS provides foundational evidence to support children and their families dealing with multiple complexities.

The lack of specialized services for children and youth with complex needs places significant strain on the entire family unit. The experience of navigating disjointed systems impacts the psychological and emotional well‐being of both the children and their families (Hanson et al. 2023). For instance, perceived social support has been identified as a moderator of post‐traumatic stress disorder (PTSD) symptoms among parents of autistic children (Masa'Deh et al. 2025). Child and family outcomes are intimately linked; one systematic review of more than 60 studies described a bidirectional relationship between parental psychological distress and child interfering behaviours (Yorke et al. 2018). These findings underscore the importance of engaging both children and their families in services that address their intersecting clinical, developmental and psychosocial needs.

Accordingly, caregiver perspectives are essential when evaluating integrated, interdisciplinary neurodevelopmental services. For those neurodivergent youth who are unable to self‐report, caregiver perspectives provide the best available proxy for understanding their experiences and outcomes. Existing evaluations of integrated developmental care programmes have focused primarily on clinicians (Stadnick et al. 2022) or caregiver experiences using questionnaires (Suen et al. 2021). Detailed caregiver descriptions of their experiences in integrated, interdisciplinary programmes are essential to identify the key components of these models and to determine which services best address unmet needs. Thus, the present study aims to explore caregiver experiences of an interdisciplinary, personalized wraparound clinical model (Ontario's ENS).

## 2 Methods

### 2.1 Researcher Characteristics and Reflexivity

V.R. is a White, cisgender, neurotypical woman. Z.O.S. is a Black, cisgender, neurotypical woman. N.D. is a White, cisgender man who is neurodivergent. The coding team reviewed their findings with G.F., a cisgender, neurotypical woman of mixed ethnicity, and M.P., a White, cisgender, neurotypical woman who works as a developmental paediatrician and has referred clients to the ENS. The broader research team included managers of the ENS programme. All members of the research team were reflexive in their interpretations, noting how their identity and lived experience impacted how they understood the meaning and intent behind the communication shared from the caregivers who engaged in the ENS.

### 2.2 Study Design and Paradigm

We followed a qualitative descriptive design (Sandelowski 2010) from a constructivist paradigm (Appleton and King 2002), recognizing that as researchers, we co‐create meaning with participants.

### 2.3 Setting

The ENS was established in 2022 across a group of three Ontario hospitals: Holland Bloorview Kids Rehabilitation Hospital (HB), Children's Hospital of Eastern Ontario (CHEO) and McMaster Children's Hospital (MCH). The ENS was designed to provide tailored wraparound, interdisciplinary care to children/youth clients and their families with co‐occurring neurodevelopmental, mental health and physical health conditions. Clients can access ENS through various pathways: internal referrals (i.e., cross‐departmental referrals within the hospital), external referrals (e.g., physicians and child welfare agencies) and family self‐referral in some regions (Rombos et al. 2026).

Services are tailored to client and family needs and can include care coordination, psychopharmacology, medical care, behaviour therapy, speech therapy, occupational therapy, psychotherapy, family therapy and respite, among others. Service models and offerings differ slightly across the ENS sites, with most of the care being provided on‐site, some provided at home and more limited care available via phone or teleconferencing.

This study was approved by the Research Ethics Board of each site (HB #671, CHEO #24/14X and MCH #17639).

### 2.4 Sampling and Recruitment

Our target sample was caregivers of children and youth clients accessing care through the ENS programme. We limited participation to caregivers whose child had a minimum of 6 months of enrolment in the programme to ensure that participants could reflect on the continuum of care experiences. Among the 29 participants, the average length of time in the programme was 18 months, with the shortest being 7 months and the longest being 32 months. Eight caregivers who were approached declined to participate in an interview. We used purposive sampling to broaden representation of caregiver race, ethnicity, child gender and socio‐economic status across the three study sites.

### 2.5 Data Collection

The semi‐structured interview guide (Appendix 1) was developed by the study team to elicit caregiver experiences across five key programme domains: family functioning, goal achievement, quality of life, service use and satisfaction. ENS clinicians reviewed the guide to ensure questions reflected programme aims, and members of the study team with methodological expertise reviewed the instrument for face validity. We piloted the interview guide with a parent of an ENS client to gauge question clarity, relevance and length.

One member of the study team (V.R.) conducted interviews with individual participants, via Zoom Healthcare (*n* = 25), in‐person (*n* = 1) or by telephone (*n* = 3) between July 2024 and December 2025. All participants provided consent prior to the interview and completed a demographic questionnaire. The 29 interviews were transcribed verbatim, and research staff subsequently reviewed the transcripts for accuracy and to anonymize the data.

### 2.6 Data Analysis

We conducted a thematic analysis (V. Braun and Clarke 2021; Braun and Clarke 2006), in keeping with the qualitative descriptive approach. Two members of the study team (V.R. and Z.O.S.) inductively coded five transcripts to develop the initial coding guide. N.D. was subsequently added as a coder, and all coders met to review two independently coded transcripts to ensure consistency. From there, independent coding of transcripts was completed by V.R., Z.O.S. and N.D., all of whom met bi‐weekly after coding two to three transcripts to discuss any additional codes and preliminary themes. All data were entered by V.R., Z.O.S. and N.D. using a qualitative analysis software, NVivo (Lumivero, Version 15). Following coding, the coding team met with the team lead (M.P.) and research manager (G.F.) to advance the analysis into themes and subthemes relevant to the study aims.

## 3 Results

A total of 29 caregiver participants (Hospital 1 *n* = 11; Hospital 2 *n* = 10; and Hospital 3 *n* = 8) were interviewed. Participants represented varied genders, racial/ethnic backgrounds, education levels and income groups (Table 1). Our analysis identified five overarching themes: (1) inadequate care experiences prior to the ENS; (2) met and unmet expectations of the ENS; (3) benefits of the ENS for the whole family; (4) the power of big and small changes; and (5) reluctant but ready to leave the programme.

**TABLE 1:** Demographics of interview participants.

	ENS caregivers/guardians (*n* = 29)
**Gender**	
Woman	24
Man	4
Other	1
**Race/ethnicity**	
Black	2
East Asian	1
Latin‐American	1
Middle Eastern	1
Mixed	3
Southeast Asian	3
White	17
Prefer not to answer	1
**Education** ^**a**^	
Some secondary (high) school	2
Secondary (high) school diploma or equivalent	5
Diploma/certificate from trade/technical school	4
Diploma/certificate from community college	6
University certificate	1
Bachelor's (university) degree or teacher's college	8
Master's degree	1
Earned doctorate	1
**Household income**	
$0–19 999	5
$20 000–39 999	6
$40 000–59 999	1
$60 000–79 999	4
$80 000–119 999	4
$120 000–$149 999	1
$150 000 or more	6
Prefer not to answer	2

^a^ *n* = 1 did not provide education.

### 3.1 Theme 1: Inadequate Care Experiences Prior to the ENS

Reflecting on life before entering the ENS, participants expressed significant frustration with navigating health, education and social systems. Inconsistent or inappropriate services and limited support created major barriers to accessing appropriate care for their child.

#### 3.1.1 Subtheme 1a: Inaccessible, Inconsistent and Unsatisfactory Services and Support

Across sites, participants described patchy experiences of receiving support from local programmes and services. They recalled challenges finding and keeping services; these burdens were in addition to challenging circumstances they were navigating at home. As Site (S)2, Participant (P)20 shared, ‘It shouldn't take four plus years, and then three trips to [emergency room] in the same week where a window gets smashed out of an observation room for somebody to maybe pay attention to you’. When they did find services, the cadence of support was ‘spotty’ (S2P12), and often ‘fizzled out’ (S1P5). S1P10 described being stuck in a loop of ‘intake interviews, intake questionnaires, more intake, more intake, more intake, more intake, more intake. More wait lists, more wait lists, more wait lists’, resulting in spending more time advocating for support than receiving it. In some cases, bridging gaps between services was felt to be the responsibility of the caregiver, rather than the system. S1P1 shared, ‘It was so challenging navigating and bridging that gap myself. On top of being a mom and a caregiver, it was basically impossible. And it left [client] with gaps in services’.

When participants were able to access services, many reported that these did not address their needs. In some cases, services were ‘all over the place’ (S2P18), and for one family, ‘nothing was done for the individual support that he needed for his behaviour’, (S3P25) with another caregiver calling the time‐limited programme they received prior to ENS ‘a load of garbage’ (S2P13). S2P18 expressed a sentiment that was common across participants: ‘I just didn't find that it was beneficial’.

#### 3.1.2 Subtheme 1b: Impacts of Inadequate Experiences

Many participants described being in crisis as they fought to find and retain services that met their needs. Some parents felt traumatized by repeatedly witnessing their children's unsafe behaviours and by the system's inability to provide meaningful help, with S1P3 stating, ‘you don't really realize what you went through. And some days I have flashbacks, and I'm realizing I have PTSD … I'm realizing how the last five years have affected me’. Similarly, S3P20 stated that their system experiences have left them feeling ‘always on guard to know that I have to fight’. Participants described moral distress of not being able to meet their children's needs, with S1P2 noting that they could ‘only do so much’. Other participants described growing cynicism with available support, such as S2P13, who reported, ‘it's so easy … to feel jaded or, to just be really cynical about services that are available, period. Because they don't actually apply to your family’.

### 3.2 Theme 2: Met and Unmet Expectations of the ENS

Participants reflected on their expectations for the ENS, including programme components, their role and outcomes for their children.

#### 3.2.1 Subtheme 2a: Expectations of the Programme Scope, Components and Timing

Participants had varied expectations coming into the programme, informed by word of mouth and their individual needs. S2P14 had high expectations for the amount of support they would receive, sharing that they were ‘hearing a lot of positive things … saying there's going to be a lot of help’. Other parents had expectations around the types of services; S1P4 said her ‘primary expectation was medication adjustment and understanding’, while S1P2 expected ENS to ‘give [client] the tools to manage her anxieties and other things that are applied to her diagnosis’. In some cases, parents' expectations were unclear, with S1P10 stating, ‘I don't know what I expected exactly’.

#### 3.2.2 Subtheme 2b: Expectations for Family Participation

Family engagement, which included bringing children to multiple appointments and/or engaging in caregiver‐mediated aspects of the programme, was a key component of the ENS. S1P4 shared, ‘They did say there is … a dose effect … So, it does work better if you come in more … If I can't come in twice a week, then [client] just doesn't get as much goals. He doesn't get as much therapy. He doesn't get as much benefit’. Many families shared that their participation was contingent on the support system around them. S2P12 said, ‘I'm a single parent but even if I wasn't, it's really hard to be involved in a lot of the stuff that they want us to do when we don't have respite care’. Likewise, S2P13 shared that family participation is ‘what you put into it’, also highlighting that the expectations in ENS can be ‘incredibly demanding on families. Like, I often have to go lie down after I'm done a session, just because it's that involved’. Notably, some participants felt the family expectations were a positive factor; for instance, S1P1 described leveraging the experience for the child to ‘start bonding with his dad’.

#### 3.2.3 Subtheme 2c: Expectations of Programme Outcomes

In several cases, participants' expectations of the ENS were built on prior service experiences. Some parents came into ENS ‘so deflated by everything already, with no huge expectations’ (S3P20), while S2P15 ‘didn't think [the ENS clinicians] were going to be able to help’. Additionally, for S2P15, the expectations around ENS being ‘publicly funded’ brought on thoughts of ‘this is not going to be much’ but found it was ‘the complete opposite’.

Several families spoke to how ENS exceeded or met their expectations, often to their surprise. S1P5 shared about feeling listened to in the programme:

I didn't expect it to be that helpful … But with this program, I felt like they really care … They were really paying attention to my concerns, and they were finding solutions together, and they were looking to help me, and I could feel it … and they did help me a lot. So, I think it was above my expectation.

S1P6 connected feelings of satisfaction with attention to their son's particular needs:

My expectations were that my son's individualized needs were going to be met and I'm coming out of the program with exactly what I expected … I'm actually exiting a program with complete satisfaction.

### 3.3 Theme 3: Benefits of the ENS for the Whole Family

Through programming that was flexible, adaptable and inclusive, the ENS wraparound approach benefited not just the child/youth but the whole family.

#### 3.3.1 Subtheme 3a: Openness to Family Needs

Participants described feeling safe and comfortable in the ENS and attributed this to the welcoming and accepting nature of the team. Many participants described this as a key difference from previous services they had accessed. They also contrasted these feelings of connection with the sense of isolation that often comes with caring for a child with complex needs. As S2P18 noted, ‘it's hard for some people to understand what we're dealing with’. S2P13 described how supported they felt during a situation at home as a clinician arrived for an appointment:

… having people sort of come in … [to] help to diffuse a situation where I was in distress, in danger, upset … And have someone just … be able to turn to me and say, like, ‘Is there anything I can do to support you right now?’ Having someone ask me that, there are people that care about our family, and I can tell. They're invested in our family.

Participants highlighted how impactful it was knowing there was someone on the team they could contact with questions or concerns. Many discussed the interpersonal attributes that made care experiences meaningful, as noted by S1P1:

There was empathy, there was active listening. There was checking in and connectivity. And I didn't feel alone in any part of the process. There was always a resource, whether it be a tool online or an individual that I can connect to. I kind of always felt supported along the way.

#### 3.3.2 Subtheme 3b: Building Rapport and Establishing Trust With Clients

Participants highlighted how the ENS team prioritized building relationships with their children while also helping them work towards their goals. S3P25 noted how clinicians emphasized making her son feel comfortable:

I felt like my son's one‐on‐one needs were actually met for the first time … And they did things that made him feel comfortable. They let him know that whatever he is comfortable with, if he needs to take a break, tell them. They'll stop talking, they'll take a break, they'll change the subject. But, like, it made him know that his voice is valid.

Even on days when clients were less engaged, clinicians listened and followed their lead. As S2P19 said, ‘They've done a really good job of forming a rapport and building bonds with him. And they've tried to keep it going when he wanes out of interest … They really do try to get them to buy in’. S1P5 noted that her child would previously ‘tap out [of a service] after six weeks’ and was willing to attend the ENS sessions because he ‘sees the benefit himself’. After repeated experiences of their children failing to connect with clinicians, many participants had given up hope for these interactions. S3P20 described a sense of relief that their child was able to connect with someone on the team:

I still get teary‐eyed. When [client] finally connected with this social worker, and he actually hugged her … just knowing that there was a connection there, and that he felt safe that he could actually open up to somebody outside about his true feelings … I can trust her to be there for him.

#### 3.3.3 Subtheme 3c: Coordinated Care That Fits Family Needs

Participants described care in the ENS as being personalized to both family and client needs, rather than a programme they needed to fit into. S2P13 described the importance of picking relevant goals with the team, noting the value of goals ‘that you actually want to work on’. Many participants talked about their time in the ENS as one where their priorities were at the core of the care they received, describing it as ‘family‐based’ (S3P27). This included listening to clients' needs and ‘meeting [client] where he's at’ (S3P27), checking in with families between visits and being ‘willing to tweak the plan of care if need be’ (S3P27). Many participants mentioned that open communication between the team, family and client improved their experiences and reduced the burden of care coordination that they previously had to assume.

It's more collaborative and very team‐oriented, which is great. Like it's nice that all the caretakers are speaking to each other. So I don't have to repeat myself a hundred different times to new therapists. – S2P16

### 3.4 Theme 4: The Power of Big and Small Changes

Participants described a range of ways that the programme affected their families. What may seem like a minor change to an outsider can have a major impact on a family managing multiple complexities.

#### 3.4.1 Subtheme 4a: Building Clients' Relationships With Themselves and Others

Participants described that after the ENS, their children were better equipped to understand and express their wants, needs and boundaries. In situations that were previously challenging, their children were now able to communicate their needs or limits. As S1P10 said, ‘he's telling me more, but also I think he's hearing me more’. Importantly, these new skills did not prioritize the adoption of neurotypical norms:

There's all this teaching of ‘how to be normal’ with these other programs … I think the program highlights that, rather than trying to mask them … It's like ‘No, you act how you act, but here's the tools that you need to get you through it. And you can be who you are’. – S1P10

Many parents shared how their relationship with their children had shifted because of their participation in ENS. They became more attuned to ‘body cues’ (S3P20) and knowing ‘when [client] needs to take time away from either different activities or people’ (S3P20). S1P3 described the value of their son's increased communication: ‘Being able to see him have these preferences and these choices, I'm able to get to know him rather than just directing him. [To] be his caregiver. I'm actually getting to make a bond with my son’.

#### 3.4.2 Subtheme 4b: Recognizing Conditions for Success

Through better understanding of their children, participants were able to reconsider their expectations and ultimately improve their participation in the community as a family. For instance, S1P1 described the importance of ‘communicating our limits and needs and accepting them’. S1P4 said, ‘we have learned to not get stuck on things that we once thought were very important’ and ‘we don't have to feel guilty for not being “on” all the time’. Through a better understanding of family‐level needs, participants set meaningful expectations and boundaries:

The biggest one for me as the parent was the boundary aspect of it. Setting ourselves up to succeed … Being realistic about what our family's capacity is. And it's okay to not want to go to this giant event, even though people are trying to get you to go, but maybe it's not for us and it's okay to say no. So that was a big one for us, letting go of that guilt. – S1P1

Other families described how reframing their expectations and recognizing their children's cues enabled family activities:

We've learned to identify the signs in her before they get to be a big problem. But also it's made us realize that there's some things that she just can't handle and we should stop putting her in those situations or we should adjust. Like going to see family, for example … we brushed it off before, didn't really understand. And now we know when she tells us something we listen and we take her out of the situation or we support her through it, if we can't [take her out]. – S2P15

#### 3.4.3 Subtheme 4c: Big‐Picture Changes for the Whole Family

Families reflected on how their involvement in ENS has impacted their whole family's quality of life, describing the programme as ‘an all‐encompassing big picture’ (S1P5). Skill acquisition was not limited to the client or even to the main caregiver(s), with S3P20 noting, ‘I think the program really has helped us as a family to open up more emotionally and to be okay with emotions’. Participants felt they were better equipped to access community‐based services, with S3P22 noting that the programme ‘changed my whole life [for the] better’ and how the team ‘helped in every area, like housing, therapy, school’. Other families noted how the support provided by the programme has helped them cope on their own without relying on emergency services as they previously did:

I think having ENS … has helped us as a family continue to survive. Because I don't know what would have happened. We haven't had to call the police or have an ER visit in quite a while [and] that's due to ENS. – S2P16

### 3.5 Theme 5: Reluctant but Ready to Leave the Programme

Families described mixed emotions transitioning out of the ENS and returning to community‐based supports.

#### 3.5.1 Subtheme 5a: Fear of Discharge

Many participants worried about being discharged from the ENS as they grew more comfortable participating in the programme, with S1P10 sharing that ‘I'm worried about what's going to happen once he loses these people that have become really important to him’. Clients and caregivers formed close bonds with clinicians, complicating the discharge process:

[Client] likes going to see them. And he wants to continue. He was very upset when he had to say goodbye. He forms attachments and because he was abandoned once, he feels like he's abandoned if they say goodbye … he thinks they don't like him anymore. – S2P18

We had to develop a strategy and a plan, to kind of let him know that his appointments with [clinician] were not going to continue on … they developed a strategy where he's gonna rename his stuffy after her so he can remember [clinician] … having that connection and that positive relationship, and that memory and then honing it into something that he could see on a regular basis, which I thought was very nice. – S1P1

After previous unsuccessful services and experiences of crisis, participants experienced fears about returning to a precarious state when new challenges arise:

I'm terrified of being discharged … I know that once we achieve one thing, something else is gonna come up … That's what scares me, is sort of the idea of you met your goal … and being scared of being left in a state of crisis. – S2P13

#### 3.5.2 Subtheme 5b: Tools for Transition

Participants understood their time in the ENS was limited and reported being supported through a gradual transition process before leaving the programme. Participants described the ENS providing them with tools that prepared them for success beyond the programme. Resources and strategies enabled families to feel comfortable and confident with the discharge plan. Some participants expressed that they were unaware of all available funding options prior to participating in the ENS. S3P21 described the value of the resources shared with them prior to leaving the ENS:

Before they discharged me, they made sure I'm connected to lots of resources that I can reach out to anytime, and with that year, I learned a lot about lots of resources that I didn't know about before. Even though I was involved with [centre] for more than 6, 7 years. But in this one year, I learned like, much more …. – S3P21

#### 3.5.3 Subtheme 5c: Gratitude for Programme

Participants frequently expressed gratitude for the services and care they received from the ENS, emphasizing how meaningful and transformative the programme has been for their overall quality of life. Families were impressed by the quality of the service; for example, S2P15 expressed feeling ‘shocked that something like this exists’. S3P29 expressed gratitude related to the programme's effect on their own well‐being: ‘I feel like I'm owing a lot of gratitude for [helping] me out of really dark places, really difficult spaces where I didn't know that I could navigate certain things on my own’. In reflecting on their feelings of gratitude, S2P13 connected this with a strong desire that the programme retain its current structure:

So when I say, that this has been truly, remarkably different. I don't say that lightly. Honest to God, [I] have a fear that these services will be removed or watered down to the point where they don't exist the way they do now. Understanding that things … when they launch as pilot programs … the idea is to change them and shape them and grow them. Oftentimes … you start big and … budget restraints and staffing restraints and volume of kids trying to get in will shake that down and whittle it down to something different. I'm honestly scared that that will happen, because the value of what this program has added to our family's life, to my child's life … it's not comparable to anything else.

## 4 Discussion

This qualitative study provides insight into the perspectives and experiences of caregivers who had a child receiving care in the multi‐site ENS programme, which is a trans‐diagnostic, wraparound, interdisciplinary service for children with multiple intersecting complexities. We identified five themes from interviews with caregivers in the programme: (1) inadequate care experiences prior to the ENS; (2) met and unmet expectations of the ENS; (3) benefits of the ENS for the whole family; (4) the power of big and small changes; and (5) reluctant but ready to leave the programme. Together, these speak to the ways that families are shaped by repeated encounters with services that fail to meet their needs, the family‐level benefits of tailored approaches and how integrated care supports families in gradually returning to community‐based services.

Given that the ENS is designed for families whose needs have not been adequately met by existing services, reports of insufficient access and low perceived value of prior care experiences in this population were anticipated. Participants described receiving inconsistent care delivery, getting stuck in a loop of administrative work and needing to fill gaps themselves. These unsuccessful attempts at accessing care are not benign, with some families in our study describing a constant sense of needing to fight for their child and others feeling cynical about any prospects of receiving help. Many of these sentiments are echoed in the existing literature; for example, perceived access to support moderates PTSD symptoms among caregivers of autistic children (Masa'Deh et al. 2025). Unsurprisingly, families of children with complex needs also experience higher rates of missed employment, poorer mental and physical health and an overall lower quality of life (Holmes et al. 2024).

Caregiver expectations coming into the ENS programme were informed by prior experiences, word of mouth and individual hopes. Most caregiver participants indicated that their expectations were met or exceeded, which was sometimes surprising to them given past experiences. Unmet expectations were tied to time limitations in the programme and concerns about what could be achieved in a particular time frame. Studies indicate that families prefer a needs‐based approach to care over a time‐based approach, particularly within a caregiver‐mediated model of therapeutic support (Fabiano et al. 2016). Families sometimes struggled with the intensity of the programme, which included frequent clinic visits and their active participation. While interventions that actively engage caregivers often report lower family stress as an outcome (Factor et al. 2019), our results suggest that individual circumstances are of paramount importance when tailoring therapeutic services for families experiencing multiple complexities.

Caregivers emphasized the importance of flexible, family‐centred care delivered by clinicians who were welcoming and accepting. Care provided through the ENS is aligned with Sinclair's model of compassion in paediatric healthcare, including key domains of beneficence, human relating, seeking to understand and attending to needs (Sinclair et al. 2021). In contrast to past experiences, participants in our study felt truly cared for by their clinical team, building their sense of self‐efficacy and contributing to positive outcomes. Previous work has similarly identified family empowerment as a significant mediator between adherence to family‐centred care and improvements in interfering behaviours (Graves and Shelton 2007). These findings suggest that, in addition to evidence‐informed, personalized therapies, the key qualities of providers are critically important in providing meaningful services to this population.

Caregivers reported how seemingly small changes, such as their child expressing their needs or learning to read their child's cues, could substantially increase their quality of life and participation. Neurodivergent children and their families report fewer out‐of‐home activities and lower engagement compared to neurotypical children (Van keer et al. 2019). Our results shed light on ways to improve community participation among families with complex needs. Client comfort and dignity were prioritized in clinical interactions, building autonomy and advocacy skills. Families in the ENS became more attuned to their children's cues and knowledgeable about their capacity in a given situation. These learnings helped them select activities with a higher chance of success and to feel less guilt when ending an engagement early. These examples demonstrate that the focus of ENS was not on ‘fixing’ the child but instead on optimizing the fit between the child, the family and the broader environment. Our results suggest the importance of individualized goal setting for families in complex situations, recognizing that what seems to be a small gain can be transformative.

Transitioning out of the ENS elicited mixed emotions among participants. Many caregivers described a sense of loss when reflecting on the relationships they had built with the ENS team. Participants frequently reported prior unhelpful care experiences alongside their fears of being discharged from the programme. Similar concerns have been documented in the literature on the transition from paediatric to adult services among neurodivergent youth, where caregivers frequently report fears related to fragmented supports and a lack of coordinated and comprehensive services (Chun et al. 2023). In the present study, these feelings were partially alleviated through the provision of information regarding available tools and funding. Members of the ENS team also took the time to prepare caregivers and clients for transition out of service through open communication and gradually decreasing the frequency of visits. Taken together, caregivers' prior experiences before entering the ENS and their apprehension about transitioning out of the programme highlight the urgent need for accessible, personalized, family‐centred services across the continuum of care, not only within intensive programmes such as the ENS.

Our study has many limitations to consider. Caregiver participants may have been more inclined to participate due to positive experiences in the programme, and these results may not reflect all caregiver experiences, including within the same family. While multiple coders analysed transcripts independently, this was mitigated through frequent meetings to discuss coding decisions and ensure consistency in theme identification. Additionally, because the ENS operates at hospitals in urban centres, our findings may have limited generalizability to rural and remote areas. These communities often require locally developed, culturally sensitive approaches to effectively meet the unique needs of families (Cermak et al. 2026).

## 5 Conclusion

Caregivers of children and youth with neurodevelopmental diagnoses, co‐occurring conditions and socio‐economic adversity face persistent challenges trying to access care programmes that match their children's and families' needs. In contrast to previous experiences, Ontario's ENS met or exceeded family expectations. Key elements of the programme included openness, adaptability, compassion and championing autonomy of the child and family. Although families expressed reluctance at the programme's conclusion, they reported feeling better equipped to manage day‐to‐day challenges and to support their children's ongoing developmental needs.

## Author Contributions

**Victoria Rombos:** conceptualization, writing – original draft, writing – review and editing. **Zainab O. Salami:** conceptualization, writing – original draft, writing – review and editing. **Nicholas Denomey:** conceptualization, writing – original draft, writing – review and editing. **Genevieve Ferguson:** conceptualization, writing – original draft, writing – review and editing. **Renee Baysarowich:** conceptualization, writing – review and editing. **Dennis Newhook:** conceptualization, writing – review and editing. **Stephanie Sutherland:** conceptualization, writing – review and editing. **Irene Drmic:** conceptualization, writing – review and editing. **Jordan Edwards:** conceptualization, writing – review and editing. **Srishti Sharma:** conceptualization, writing – review and editing. **Kathryn Decker:** conceptualization, writing – review and editing. **Tamara Milicevic:** conceptualization, writing – review and editing. **Melanie Penner:** conceptualization, writing – original draft, writing – review and editing.

## Funding

The authors declared financial support was received for this work and/or its publication. The ENS is supported by joint funding through Ontario Health, via the Ministry of Health and the Ministry of Children, Communities and Social Services.

## Ethics Statement

This study was approved by the research ethics board of each site.

## Conflicts of Interest

M.P. has done paid consulting for the following (unrelated to this publication): Addis & Associates, the Province of Nova Scotia and MedCounsel. The remaining authors declared that this work was conducted in the absence of any commercial or financial relationships that could be construed as a potential conflict of interest. Many of the authors who contributed to this work hold clinical, leadership or evaluation roles within the ENS and/or work closely with the clinical teams at their respective sites.

## Data Availability

The original contributions presented in the study are included in the article. Further inquiries can be directed to the corresponding authors.

## Appendix 1 EN Interview Guide

Questions for families

Today I will be talking to you about your experience as a parent/caregiver of a child that was involved in the Extensive Needs (EN) program. Your experience will be used to help inform how the program is delivered in the future. This interview is voluntary and you can stop the interview at any time or skip any questions you do not want to answer. No one outside the evaluation team will have access to your individual interview information. This means that your care team will not know what you say during this interview. Information that is shared will be de‐identified, meaning it will not be linked to your name or your child's name. Do you have any questions about this part? The interview will be recorded so that it can be transcribed. Is this okay?

So now we will begin talking to you about your experience as a parent/caregiver of a child that was involved in the EN program. As you may know, the goal of EN is to provide integrated care to meet needs that were not being met by current services, as well as to build skills within your family and strengthen connection to community support. Your thoughts and feedback about your experience will be used to inform the delivery of the program moving forward. This interview will be about an hour long. Do you have any questions before we begin?

1 How, if at all, is the care you have received through the EN program different from the care you have received in the past? (Probe for specific examples about the program, what's different/unique, whether these differences were good or not)
2 How did your level of involvement with this program differ from others (programs)? (*List to prompt)*
  - Being an active participant in your child's treatment and care
  - Number of visits/length of sessions
  - Professionals seen

What did you like about the programme?

3 What did not like? Is there anything that you would like to see added to the programme? (ex. available supports, other services, and the availability of those services/supports, wait times)
4 Has EN made a change in your child's (the client) life? If so, how and in what areas? *(List to prompt)*
  - Home environment (routines, transitions, conflicts, meal times, etc.)
  - Child's participation in activities, school, community, etc.
  - Transferability of skills learnt (family members & client)

5. a) If EN **did not** make a change, why do you think this is? (Probe for reasoning behind lack of change using previous prompts)

5 What was it like for you and your family to take part in the EN program? (Probe for who was taking client to visits, most involved in care, etc.)
6 Has EN made a change in your (parent/guardian & siblings) overall health and wellbeing?
  - Parent/Guardian's participation in work, activities, school, community, family outings, religious services, etc.
  - Sibling's participation in activities, school, community, etc.

What were your expectations going into the EN program? Did your child get what they needed out of the program? Why or why not? (Probe for what they needed from the program)

7 Was there a pivotal/stand‐out/special moment that you remember?
8 Did the program help you connect with other community service providers?
  - During your time in EN
  - After leaving EN (for discharged families ONLY)

Is there anything else you would like to add about your experience of the EN program?

Questions for families that have been discharged:

9 Can you tell me about your experience leaving (transitioning out of, being discharged) from the EN program?
